# Outcome of pediatric procedural sedation & analgesia in a tertiary care hospital in Pakistan

**DOI:** 10.12669/pjms.316.8607

**Published:** 2015

**Authors:** Humaira Jurair, Amyna Bhimani, Anwar-ul-Haque

**Affiliations:** 1 Humaira Jurair, FCPS, Senior Instructor, Department of Pediatrics & Child Health, Aga Khan University Hospital, Karachi, Pakistan; 2 Amyna Bhimani, Diploma in Nursing, Registered Nurse, Department of Nursing Service, Aga Khan University Hospital, Karachi, Pakistan; 3 Anwar-ul-Haque, MD, FAAP, Associate Professor, Department of Pediatrics & Child Health, Aga Khan University Hospital, Karachi, Pakistan

**Keywords:** Procedural sedation & analgesia, Pediatric, Safety

## Abstract

**Background and Objective:**

Procedural sedation and analgesia (PSA) is pharmacologically induced state which allows patients to tolerate painful procedures while maintaining protective reflexes. It is the standard of care but there is limited data from Pakistan. Our objective was to assess the safety of the procedural sedation and analgesia in pediatric population at a tertiary care setting.

**Methods:**

A retrospective notes and record review was conducted at the Aga Khan University Hospital, Karachi over 4 years from April 2010 to August 2014. Patients were between ages 6 months to 16 years and were in low risk category. The combination of Ketamine and Propofol were used. Data collected on the standardized hospital PSA form. All procedures were performed by two trained persons.

**Results:**

A total of 3489 diagnostic and therapeutic procedures were performed. Satisfactory level of sedation was achieved for 3486 (99%) of procedures. Adverse events occurred in 21 (0.6%) patients including: 12 (0.3%) episodes of hypoxia, 07 (0.2%) episodes of apnea, 02 (0.06%) episodes of post sedation hallucination. No major events were noted.

**Conclusion:**

Procedural sedation & analgesia for children using Propofol and Ketamine is found safe and effective in our setting.

## INTRODUCTION

With increasing number of diagnostic and therapeutic procedures being performed on children and increased awareness about the presence of procedure related anxiety and pain, the demand for sedation and analgesia is increasing. Main aim of Procedural sedation and analgesia (PSA) is to reduce fear and anxiety of child, obtain their cooperation, induce unawareness and achieve immobilization to allow a necessary procedure to be performed, while keeping the child safe.^1^ PSA is well established medical discipline in developed countries. There is however, limited data from Pakistan. We carried out this hospital based study to enhance our understanding about optimal application and safety of PSA in the local population.

## METHODS

Study conducted at the Aga Khan University Hospital, Karachi after obtaining institutional ethics committee review board’s approval. Retrospective chart review was done over a period of 4 years from April 2010 to August 2014. Study population included children between ages 6 month to 16 years who received PSA for various indications. PSA was performed at various locations outside the operating room environment within the hospital. All procedures were performed in accordance with American society of Anaesthesiology guidelines (ASA).^1^ Patients with ASA physical status Class I and II who are considered suitable candidates for PSA were included in study.^1^,^2^ Informed written consent was obtained from parents before each procedure. Sedation team comprised of an intensive care physician and a trained sedation nurse. Physician typically oversaw drug administration, while the assistant nurse continuously monitored the patient for complications and documented all the relevant information.

Two drug combinations was used for all procedure; Ketamine & Propofol. Ketamine was given in dose of 0.5-1 mg/kg via slow IV push. This was followed by intra-venous Propofol initially in the dose of 1-2 mg/kg titrated till the patient was sedated. Pulse oximeter was attached and oxygen was administered in all cases. The procedure was initiated with monitoring of patient throughout the procedure and until complete recovery from effects of sedation. A PSA form was used to record pertinent clinical and demographic characteristics of patients, information related to the procedure, vital signs and the occurrence of complications. Simple descriptive statistics were used.

### Definition & Outcomes

### Success of sedation

defined as successful completion of the procedure.

### Sedation failure

defined as inability to achieve adequate sedation with optimal drug dosage

### Complications were defined as

- ***Apnea:*** Temporary suspension of breathing for greater than 20 second duration
- ***Hypoxia:*** Sustained pulse oximetry saturation <90% for greater than 1 minute duration
- ***Cardiac Arrest:*** Requirement of Cardiopulmonary resuscitation
- ***Hallucination or emergence reactions:*** Perception in the absence of external stimulus like seeing movement or hearing noises, a loss of sense of time and orientation
- ***Allergic Reactions:*** Presence of rashes, itching, flushing, facial redness, respiratory difficulty or abdominal pain
- Vomiting

## RESULTS

During 4 years, 3489 diagnostic and therapeutic procedures were performed using PSA in 3233 children. 2199 (68 %) patients were male. Among age category 26 patients (0.8%) were < 1 year of age, 1127 (34.8 %) were between 1 year to 5 years and 2080 (64.3 %) were between 6 to 16 years old. Descriptive characteristics and indications for PSA are mentioned in Table-I and II respectively. Most common indication was oncological procedures. Satisfactory level of sedation was achieved for 3486 (99%) of the procedures. Sedation failure occurred only in 3 (0.08 %) patients. Adverse events occurred in 21 (0.6 %) patients, including: 12 (0.3%) episodes of hypoxia, 7 (0.2%) episodes of apnea and 2 (0.06%) episodes of post sedation hallucination. There were no major events, no recorded episodes of cardiac arrest or other emergencies requiring endotracheal intubation. Complications were managed with simple interventions, like hypoxia resolved after re-positioning of airways and increasing oxygen inhalation. Apneic episodes required brief bag and mask ventilation. Hallucination resolved after child became fully awake.

**Table-I:** Descriptive characteristics of Procedural Sedation & Analgesia.

Characteristics	Number / (%)
Total no of patients	3233
*Gender*	
Male	2199 (68)
Female	1034 (32)
*Age*	
< 1 year	26 (0.8)
1-5 year	1127 (34.8)
6-16 year	2080 (64.3)
Total no. of procedures	3489
*Procedural Area*	
Pediatrics ward procedure room	2614 (74.9)
Daycare oncology unit	745 (21.3)
Radiology department	107 (3.06)
Neurophysiology department	19 (0.54)
Endoscopy suite	04 (0.11)
Successful procedure	3486 (99)
Sedation failure	03 (0.08)
Adverse effects	21 (0.60)

**Table-II:** Indications of Procedural Sedation and Analgesia.

	Indications	Number
1	Intra-thecal chemotherapy administration	2280
2	Bone marrow aspiration, biopsy	781
3	Lumber Puncture	126
4	PICC line, CVP line, Joe Cathe insertion	101
5	Wound dressings	66
6	Ultrasound guided Liver, Renal, abdominal mass Biopsy	28
7	Chest drain removal	24
8	Botox injection administration	16
9	CT scan guided procedures	13
10	BERA test	12
11	Ultrasound guided abdominal drain insertion, ascitic tap	10
12	Various Laceration repair	10
13	EMG and nerve conduction study	06
14	Various abscess drainage	05
15	Upper, Lower GI Endoscopy	04
16	Various Orthopedic procedures	04
17	Skin biopsy	02
18	SSEP test	01

## DISCUSSION

PSA is an evolving field in patient care, with increasing demand for performance of diagnostic and therapeutic procedures in safer and pain and anxiety free environment.^1^,^3^ Advantages include involvement of non-specialist care providers (non-anaesthetists), performance outside operation theatres thereby saving cost, and reduction of time-to-treatment.

Different medications or combinations can be adopted for provision of PSA. In our setting we used the combination of Propofol and Ketamine because of the synergistic effects of these medications with each other and because of reduction in dose requirement as demonstrated by several studies.^4^ Additional advantage of this combination strategy is reduction in adverse risks associated with either Propofol and Ketamine. For example hypotension and respiratory depression caused by Propofol can be reduced with increases in circulatory catecholamine surge induced by ketamine. Similarly, nausea and emergence reactions associated with Ketamine can be decreased by the antiemetic and anxiolytic properties of Propofol. Multiple studies have supported this practice.^4^-^6^

Safety is, of course, the utmost concern. Patients with ASA class I and II are considered appropriate for PSA.^1^,^2^ As it is difficult to predict how an individual child will respond to a specific medication, the practitioner must be able to manage potential complications.^2^ In our setting PSA providers have resuscitation and advanced paediatric life support skills, as well as specific training in paediatric procedural sedation.

Failed or inadequate sedation can lead to patient and parental anxiety, procedure cancellation, possible delays in diagnosis, treatment and financial losses. The reported incidence of inadequate or failed sedation ranges between 3 – 16 %.^2^,^7^ In our setting, sedation failure was negligible (0.08 %) patients.

Multiple large studies have shown that when used properly, drugs used for PSA have low rates of complications, manageable with simple manoeuvres. Reported adverse events range from 0.6% to 7.8%.^2^,^7^,^10^. Adverse events encountered in our study were 0.6%. These were mostly respiratory adverse events; appear to be consistent with other published studies.^2^

## CONCLUSION

Safe and effective sedation and analgesia is a critical skill which requires expertise and competency in training. Currently only few centres of Pakistan are utilizing this service. Our data suggests that PSA is safe and acceptable, given that stringent standards of care are met and established protocols are strictly followed. Minimal complication rates and successful achievement of sedation and analgesia shown by the record review may encourage other clinicians to adopt PSA in their setting.
